# Hepatic glycogen synthase (GYS2) deficiency: seven novel patients and seven novel variants

**DOI:** 10.1002/jmd2.12082

**Published:** 2020-02-25

**Authors:** Elena A. Kamenets, Elena A. Gusarova, Natalia V. Milovanova, Yulia S. Itkis, Tatiana V. Strokova, Maria A. Melikyan, Irina V. Garyaeva, Irina G. Rybkina, Natalia V. Nikitina, Ekaterina Y. Zakharova

**Affiliations:** ^1^ Research Centre for Medical Genetics Federal State Budgetary Institution Moscow Russia; ^2^ Federal Research Center for Nutrition and Biotechnology Federal State Budgetary Institution Moscow Russia; ^3^ Pirogov Russian National Research Medical University Federal State Budgetary Institution Moscow Russia; ^4^ National Research Center for Endocrinology Federal State Budgetary Institution Moscow Russia; ^5^ Morozov municipal children's Hospital of Moscow City Federal State Budgetary Institution Moscow Russia; ^6^ Clinical‐Diagnostic Center of Mother and Child Health Protection Federal State Budgetary Institution Yekaterinburg Russia

**Keywords:** glycogen storage disease, glycogen synthase, GSD0, GYS2, hypoglycemia, ketones, next generation sequencing

## Abstract

Glycogen storage disease type 0 (GSD 0) is an autosomal recessive disorder of glycogen metabolism caused by mutations in the *GYS2* gene manifesting in infancy or early childhood and characterized by ketotic hypoglycemia after prolonged fasting, and postprandial hyperglycemia and hyperlactatemia. GSD 0 is a rare form of hepatic glycogen storage disease with less than 30 reported patients in the literature so far.

DNA samples of 93 Russian patients with clinical diagnoses of hepatic GSDs were collected and analyzed by next‐generation sequencing custom target panel and by direct sequencing. Seven new GSD 0 patients with variable phenotypes were found showing 10 variants. Seven variants are novel.

We present seven new GSD 0 patients with variable phenotypes. Overall, 10 different mutant alleles of the *GYS2* gene were found. Seven of them are novel: c.214delC, c.845delT, c.1644C>A, c.205T>A, c.929G>T, c.1169G>C and c.1703C>A. Three of the novel variants were annotated as pathogenic and likely pathogenic; four other variants have an uncertain significance.

The current results expand the spectrum of known mutations in *GYS2* and suggest that phenotypes of GSD 0 are more variable and less specific than the reported ones.

**Synopsis:**

Seven new patients with glycogen storage disease type 0 were found using next‐generation sequencing and seven novel variants of *GYS2* gene were annotated.

## 1 INTRODUCTION

Glycogen storage disease type 0 (GSD 0, MIM 240600), also known as aglycogenosis, is an autosomal recessive disease caused by genetic defects in glycogen synthase. The overall frequency of GSDs is approximately 1/25000‐1/20000 newborns. GSD 0 is an extremely rare form, representing less than 1% of all GSDs with less than 30 cases reported since the discovery of the disease in 1963.^1^, ^2^ Depending on the enzyme location, GSD 0 can be subdivided into liver and muscle types.^2^, ^3^

Hepatic glycogen synthase is encoded by *GYS2* (*MIM 138571*) located on 12p12.2 and contains 16 exons spanning over 30 kb of genomic DNA.^4^, ^5^ It consists of 703 amino acid residues. Twenty‐two deleterious mutations in *GYS2* have been described (http://www.hgmd.cf.ac.uk/ac/index.php).

Mutations in *GYS2* lead to impaired glycogen synthesis. In contrast to the other GSDs that are associated with glycogen accumulation, GSD 0 is characterized by an extremely low amount of glycogen in hepatocytes. The disorder is characterized by hyperketotic hypoglycemia without hyperlactatemia after fasting. Postprandial hyperglycemia and hyperlactatemia develop due to the conversion of meal‐derived carbohydrates to lactate, as glucose cannot be converted to glycogen. Fasting hypoglycemia usually manifests in late infancy when overnight feedings are stopped. Children may exhibit early morning drowsiness, pallor, vomiting and fatigue. In more severe cases impairment of consciousness occurs. Seizures associated with hypoglycemia are uncommon. Unaffected gluconeogenesis and fatty acid oxidation processes counteract the decrease of blood glucose level, and the subsequent increase of ketones in the plasma provides the brain with alternative fuel (3, 6). Therefore, in general, GSD 0 does not impact mental development. Short stature and osteopenia are common features (1, 7).

Because of the very low numbers of reported patients some specialists consider this disorder to be under‐diagnosed (1, 3, 6, 8, 9). Apart from the classical clinical manifestation, GSD 0 may be very mild (drowsiness, lack of attention, pallor and disorientation before the first meal of the day) or proceed asymptomatically, and therefore can be missed.

## 2 MATERIALS AND METHODS

### 2.1 Patients

DNA samples of 93 Russian patients with a clinical diagnosis of hepatic GSD were collected and analyzed by next‐generation sequencing using a custom target panel and by direct sequencing in the Research Centre for Medical Genetics of Moscow from January 2016 until December 2017. Age of the patients varied from 0 to 16 years (median 3.5 years). The inclusion criteria were hypoglycemic episodes (glucose blood level < 3.3 mmol/L) or/and liver enlargement with or without cytolysis. In all these patients, GSDIa and GSDIb were previously excluded by direct sequencing of the corresponding genes.

The diagnosis of GSDs was suspected based on clinical features, routine biochemical tests, 180 minute glucose tolerance test, 24‐hour glucose monitoring and ultrasound examination of the abdomen.

DNA was extracted from peripheral blood using the DNAPrep100 Kit (IsoGene, Moscow, Russia) according to the manufacturers’ recommendations and stored at –20°C prior to analysis.

### 2.2 Sequencing

Next‐generation sequencing (NGS) was performed using the Ion S5 system (Thermo Fisher Scientific). A custom panel, compiled in Ion AmpliSeq Designer, covered transcribed sequences of 47 genes (exons and UTR regions) associated with hepatic pathology, in particular the following 12 genes encoding hepatic proteins of glycogen metabolism: *AGL, G6PC, GAA, GBE1, GYS2, PFKM, PGAM2, PHKA2, PHKB, PHKG, PYGL, SLC37A4*.

Sequencing reads were aligned to the NCBI human reference genome (GRCh37/hg19). The obtained results were analyzed using Torrent Suite software. ANNOVAR software was used for variants annotation. Visual analysis of the data and manual filtering of artifacts were performed using Integrative Genomics Viewer.

Sanger sequencing with previous amplification of target gene fragments by PCR was used to verify detected single nucleotide variants. All PCR fragments were subsequently sequenced on ABI 3500xL Genetic Analyser system (Thermo Fisher Scientific). Analysis of Sanger sequencing data was performed using Chromas software and Nucleotide‐BLAST software.

In silico analysis of the functional impact of novel missense variants was performed using the predictive algorithms MutationTaster, PolyPhen2, SIFT, and PROVEAN.

## 3 RESULTS AND DISCUSSION

In 58 of 93 patients different GSDs were genetically confirmed. The GSD IXa was the most frequent and confirmed in 22 patients (23.7%).

In seven patients from six unrelated families (four males and three females from 2 to 6 years old) alterations of the gene *GYS2* (NM_021957) were found.

Overall, 10 different mutant alleles in *GYS2* were identified. Three missense variants: c.116A>G:p.(Asn39Ser), c.1015G>C:p.(Ala339Pro) and c.1553A>C:p.(Glu518Ala) have been described and are classified in the Human Genetic Mutation Database (HGMD) as pathogenic (6, 10, 11). The other seven variants are novel: one frameshift deletion, two nonsense variants, and four missense substitutions (Table 1). Two variants (01) and (06) were found in the homozygous state in two patients, respectively, the other eight variants are found in the compound‐heterozygous state. The analysis of parental segregation was performed with the exception of one family (for the genotypes see Table 2). Predictive algorithms were applied to assess the significance of the variants.

**Table 1:** Annotations of the detected variants of GYS2 gene in the patients

Variant cipher	Variant	Exon	Mutation type	HGMD ID	dbSNP ID	Population frequency (ExAC)	Configuration	Mutation Taster	PolyPhen‐2	SIFT	PROVEAN	Variant classification
01	c.116A>G p.(Asn39Ser)	1	missense	CM980964	rs121918423	0.000025	Trans	pathogenic	possibly damaging	damaging	deleterious	Likely pathogenic
02	c.205T>A p.(Tyr69Asn)	2	missense	no	no	NA	Trans	pathogenic	benign/possibly damaging	damaging	deleterious	Uncertain significance
03	c.214delC p.(His72Ilefs*2)	2	frame‐shift deletion	no	no	NA	Trans	pathogenic	NA	NA	NA	Pathogenic
04	c.845delT p.(Leu282*)	6	nonsense	no	no	NA	NA	pathogenic	NA	NA	NA	Likely pathogenic
05	c.929G>T p.(Gly310Val)	6	missense	no	rs1024361436	0.000008	Trans	pathogenic	probably damaging	damaging	deleterious	Uncertain significance
06	c.1015G>C p.(Ala339Pro)	7	missense	CM980966	rs121918421	0.000025	Trans	pathogenic	probably damaging	damaging	deleterious	Likely pathogenic
07	c.1169G>C p.(Trp390Ser)	8	missense	no	rs762667150	0.000008	NA	pathogenic	possibly damaging	neutral	tolerated	Uncertain significance
08	c.1553A>C p.(Glu518Ala)	13	missense	CM177641	rs150433001	0.0029	Trans	pathogenic	probably damaging	damaging	deleterious	Uncertain significance
09	c.1644C>A p.(Tyr548*)	13	nonsense	no	no	NA	Trans	pathogenic	NA	NA	NA	Pathogenic
10	c.1703C>A p.(Thr568Asn)	14	missense	no	no	NA	Trans	pathogenic	probably damaging	damaging	deleterious	Uncertain significance

**Table 2:** Main characteristics of the patients with GSD 0

Patient №	Genotype	Sex	Age of onset	Age of diagnosis confirmation	Lowest registered glycemia, mmol/L	Glucose fasting/postprandial, mmol/L	Lactate fasting/postprandial, mmol/L	Total cholesterol, mmol/L	Urine ketones	Special aspects of the disease course
P1	c.[1015G>C];[1015G>C]	M	2y 8 m	5y	0.3	0.3/6.3	0.7/5.1	3.97	+	Growth retardation (SDS of growth = −2,87, SDS of weight = −2,39), generalized seizures
P2	c.[929G>T];[1553A>C]	F	<1 m	5y	1.8	2.2/7.5	1.15/4.96	6.6	+	Hypoglycemic episodes accompanied by vomiting starting from neonatal period.
P3	c.[205T>A];[1703C>A]	F	2y 9 m	4y	1.4	2.69/8.5	1.89/4.5	5.76	++	Digestive tract disease, alimentary adiposity, enlarged liver (2–3 cm under the edge of the rib cage)
P4	c.[116A>G];[116A>G]	F	2y 8 m	4y	1.7	3.0/8.0	1.21/3.1	4.7	++	Asymptomatic hypoglycemia
P5	c.[214delC];[1644C>A]	M	4y 6 m	4y 6 m	1.3	2.42/7.93	1.3/5.0	4.6	−	Hypoglycemia, abdominal pain, tremor, fatigue, enlarged liver (1–2 cm under the edge of the rib cage)
P6	c.[214delC];[1644C>A]	M	2y	2y	1.9	1.93/7.71	1.86/5.03	4.31	−	Asymptomatic hypoglycemia
P7	c.[845delT(;)1169G>C]	M	11 m	6y	0.8	2.3/5.0	2.19/7.28	4.2	−	Seizures, elevation of insulin level up to 3,3 μU/mL at the hypoglycemic background (normal rate < 2 μU/mL)

### 3.1 Described variants

The variants c.116A>G (01) and c.1015G>C (06) were described as disease causing by Orcho et al. Their deleterious effect was determined by analysis of the mutant glycogen synthase activity.^9^ Both allele frequencies were given as 0.000025 in the ExAC database. With the parental segregation analysis of our patients these variants can be considered as likely pathogenic according to the “Guidelines for the interpretation of sequence variants”.^10^

The variant c.1553A>C (08) was described as likely pathogenic by Ghosh et al.^11^ It was classified as damaging in the HGMD (CM177641) and estimated as probably damaging by the predicted algorithms. But there is no data about functional analysis of this allele and there were 349 carriers of this substitution given in the ExAC database, with 3 of them being homozygous. These data do not allow to consider the variant (08) as definitely disease causing. We can assume that this missense substitution produces a very slight effect on the enzyme activity and impacts the phenotype only in compound‐heterozygous state with a more deleterious allele.

### 3.2 Novel variants

The variant c.214delC (03) is a frame‐shift deletion and the variants c.845delT (04) and c.1644C>A (09) are nonsense. These alterations cause a complete absence of the gene product by lack of transcription or nonsense‐mediated decay of an altered transcript (so‐call LOF‐variants) and, according to the ExAc database, they are absent in the healthy cohort of European population. In addition, variants (03) and (09) reside in trans‐configuration subsequent to the results of the parental segregation analysis. Therefore, variants (03) and (09) should be considered as pathogenic and variant (04) is likely pathogenic.^10^

The missense variants c.205T>A (02), c.929G>T (05), c.1169G>C (07) and c.1703C>A (10) have an uncertain clinical significance. In accordance to the ExAc database, the frequencies of the variants (05) and (07) in the healthy cohort are extremely low (0.000008) and the variants (02) and (10) are absent, but there are no “strong” and not enough “moderate” and “supporting” criteria for interpreting these variants as pathogenic or likely pathogenic mutations.^10^ Different predictive algorithms interpret them in different ways (see Table 1).

### 3.3 Clinical aspects

As is seen from Table 2, patients P1 and P4 are homozygous for likely pathogenic *GYS2* variants and brothers P5 and P6 are compound‐heterozygous for pathogenic variants. In these four cases, we assumed that the genetic diagnosis of GSD 0 is confirmed. However, patients P2 and P3 are compound‐heterozygous by variants with uncertain significance and the genetic findings in patient P7 are unclear: the likely pathogenic allele (04) and the unclear allele (07) with unknown family segregation. Thus, we present three genetically confirmed GSD 0 patients and four patients with clinical symptoms of GSD 0 and with uncertain genetic data.

In five patients, the clinical diagnosis of GSD 0 preceded the genetic analysis and in the family of patients P5 and P6 the genetic diagnosis was confirmed before the clinical examination.

The clinical and biochemical data of the patients are summarized in Table 2.

All patients showed hypoglycemia after fasting (≥ 3 hours after the last feeding). The minimal registered glucose level in blood varied from 0.3 mmol/L to 1.9 mmol/L. In patients P1 and P7 hypoglycemia was accompanied by seizures.

In one of our patients, the hypoglycemic syndrome started in the neonatal period, which is unusual for all glycogen storage diseases and GSD 0 in particular. It is important to pay attention to the patients when asymptomatic hypoglycemia is associated with very low blood glucose and results in neurological symptoms like seizures and different level of decreased consciousness.

High urine and blood levels of ketones were observed in patients P1‐P4. Patients P5‐P7 had only ketonemia, with negative qualitative tests for ketones in urine. GSD 0 is one of the ketotic forms of GSDs, which present with elevated ketones, hypoglycemia and normal hormone concentrations.^12^ Ketonuria is not constant and measurement of ketones in blood is preferable.

There were also seven children referred for DNA testing with ketotic hypoglycemia in our cohort of 93 patients. In three of them, pathogenic alleles in the gene *PHKA2* in the hemizygous state were detected and, in the other four no significance changes in the 47 investigated genes were found. Therefore, we found a genetic explanation of ketotic hypoglycemia in 10 of 14 patients. According to the literature, the frequency of GSD 0 in patients with isolated ketotic hypoglycemia is about 2%.^12^, ^13^ In our group, the inclusion criteria were stricter and most of our patients had persisting hypoglycemia.

All patients P1‐P7 showed highly elevated glucose and lactate levels 3 hours after feeding (postprandial hyperglycemia >5.0 mmol/L and hyperlactatemia >1.7 mmol/L) and normal serum transaminase levels (see Table 2), which is characteristic for GSD 0.^3^, ^12^

The clinical and laboratory findings of GSD 0 are variable, and there are even nearly asymptomatic patients. The time between the first symptoms and the diagnosis confirmation has ranged from 1 to 5 years, which illustrates the difficulty of diagnosing GSD 0.

Despite the absence of glycogen storage, mild liver enlargement, not considered as a symptom of GSD 0, was observed in patients P3 and P5 (respectively, 2‐3 cm and 1‐2 cm).

At the same time, the majority of our genetic findings are undescribed variants. Four of them are missense substitutions with unclear significance. This classification requires further studies. Concerning the unclear clinical significance of *GYS2* variants in patients P2, P3, and P7 we suspect that based on genetic and clinical data corresponding to GSD0 the diagnosis could be confirmed.

## 4 CONCLUSION

The current results expand the spectrum of known mutations in *GYS2* and suggest that phenotypes of GSD 0 are more variable and less specific than previously reported.

## CONFLICT OF INTEREST

The authors declare no potential conflict of interest.

## AUTHORS CONTRIBUTIONS

E.A.K. designed the experiment, conducted the work, collected samples, analyzed and interpreted sequencing results, analyzed parental segregation, drafted the article, reported the work and is the guarantor. E.A.G. analyzed and interpreted sequencing results, analyzed parental segregation, and drafted the article. N.V.M. contributed to next‐generation sequencing and revised the article. Y.S.I. contributed to next‐generation sequencing and revised the article. T.V.S. contributed to clinical examination, collected and interpreted the clinical and laboratory data, and drafted the article. M.A.M. contributed to clinical examination, collected and interpreted the clinical and laboratory data, and revised the article. I.V.G. contributed to clinical examination, collected and interpreted the clinical and laboratory data, and revised the article. I.G.R. contributed to clinical examination, collected and interpreted the clinical and laboratory data, and revised the article. N.V.N. contributed to clinical examination, collected and interpreted the clinical and laboratory data, and revised the article. E.Y.Z. designed the experiment, conducted the work, and revised the article.

## INFORMED CONSENT

All procedures followed were in accordance with the ethical standards of the responsible committee on human experimentation (institutional and national) and with the Helsinki Declaration of 1975, as revised in 2000 (5). Informed consent was obtained from all patients for being included in the study. Proof that informed consent was obtained and is available upon request.

## References

[1] Kasapkara ÇS , Aycan Z , Açoğlu E , Senel S , Oguz MM , Ceylaner S . The variable clinical phenotype of three patients with hepatic glycogen synthase deficiency. J Pediatr Endocrinol Metab. 2017;30(4):459‐462. 28245189 10.1515/jpem-2016-0317

[2] Raghuveer TS , Gardg U . Inborn errors of metabolism in infancy and early childhood: an update. Am Fam Physician. 2006;11:1981‐1990. 16770930

[3] Weinstein DA , Correia CE , Saunders AC , Wolfsdorf JI . Hepatic glycogen synthase deficiency: an infrequently recognized cause of ketotic hypoglycemia. Mol Genet Metab. 2006;87(4):284‐288. 16337419 10.1016/j.ymgme.2005.10.006 PMC1474809

[4] Bachrach BE , Weinstein DA , Orho‐Melander M . Glycogen synthase deficiency (glycogen storage disease type 0) presenting with hyperglycemia and glucosuria: report of three new mutations. J Pediatr. 2002;140(6):781‐783. 12072888 10.1067/mpd.2002.124317

[5] Nuttall FQ , Gannon MC , Kubic VL , Hoyt KJ (1994) The liver glycogen synthase isozyme gene is located on the short arm of chromosome 12. Genomics 19:404–5 (PubMed 8188280) 8188280 10.1006/geno.1994.1086

[6] Bhattacharya K . Investigation and management of the hepatic glycogen storage diseases. Transl Pediatr. 2015;4(3):240‐248. 26835382 10.3978/j.issn.2224-4336.2015.04.07 PMC4729058

[7] Heller S , Worona L , Consuelo A . Nutritional therapy for glycogen storage diseases. J Pediatr Gastroenterol Nutrition. 2008;47:15‐21. 10.1097/MPG.0b013e3181818ea5 18667910

[8] Gitzelmann R , Spycher MA , Feil G , et al. Liver glycogen synthase deficiency: a rarely diagnosed entity. Eur J Pediatr. 1996;155(7):561‐567. 8831078 10.1007/BF01957905

[9] Orho M , Bosshard NU , Buist NR , et al. Mutations in the liver glycogen synthase gene in children with hypoglycemia due to glycogen storage disease type 0. J Clin Invest. 1998;102(3):507‐515. 9691087 10.1172/JCI2890 PMC508911

[10] Richards S , Aziz N , Bale S , et al. Standards and guidelines for the interpretation of sequence variants: a joint consensus recommendation of the American College of Medical Genetics and Genomics and the Association for Molecular Pathology. Genet Med. 2015;17(5):405‐424. 25741868 10.1038/gim.2015.30 PMC4544753

[11] Ghosh A , Schlecht H , Heptinstall LE , et al. Diagnosing childhood‐onset inborn errors of metabolism by next‐generation sequencing. Arch Dis Child. 2017;0:1‐11. 10.1136/archdischild-2017-312738 28468868

[12] Brown LM , Corrado MM , van der Ende RM , et al. Evaluation of glycogen storage disease as a cause of ketotic hypoglycemia in children. J Inherit Metab Dis. 2015;38(3):489‐493. 25070466 10.1007/s10545-014-9744-1

[13] Nessa A , Kumaran A , Kirk R , Dalton A , et al. Mutation al analysis of the GYS2 gene in patients diagnosed with ketotic hypoglycaemia. J Pediatr Endocrinol Metab. 2012;25(9–10):963‐967. 23426827 10.1515/jpem-2012-0165

